# Protein tyrosine phosphatase non-receptor type 2 as the therapeutic target of atherosclerotic diseases: past, present and future

**DOI:** 10.3389/fphar.2023.1219690

**Published:** 2023-08-21

**Authors:** Xiao-Er Tang, Ya-Qiong Cheng, Chao-Ke Tang

**Affiliations:** ^1^ Department of Pathophysiology, Shaoyang University, Shaoyang, Hunan, China; ^2^ Institute of Cardiovascular Research, Key Laboratory for Atherosclerology of Hunan Province, Hunan Province Cooperative Innovation Center for Molecular Target New Drug Study, University of South China, Hengyang, Hunan, China

**Keywords:** cardiovascular disease, atherosclerosis, PTPN2, inflammatory response, inflammatory cytokines

## Abstract

Tyrosine-protein phosphatase non-receptor type 2(PTPN2), an important member of the protein tyrosine phosphatase family, can regulate various signaling pathways and biological processes by dephosphorylating receptor protein tyrosine kinases. Accumulating evidence has demonstrated that PTPN2 is involved in the occurrence and development of atherosclerotic cardiovascular disease. Recently, it has been reported that PTPN2 exerts an anti-atherosclerotic effect by regulating vascular endothelial injury, monocyte proliferation and migration, macrophage polarization, T cell polarization, autophagy, pyroptosis, and insulin resistance. In this review, we summarize the latest findings on the role of PTPN2 in the pathogenesis of atherosclerosis to provide a rationale for better future research and therapeutic interventions.

## 1 Introduction

Cardiovascular disease (CVD) has gradually developed into a common disease threatening human health. The number of people dying from CVD ranks first among all causes of death worldwide every year (Barquera et al., 2015). Atherosclerosis (As) is the pathological basis of most CVD such as coronary heart disease, cerebral infarction, and peripheral vascular disease (Ouimet et al., 2019). As is a chronic vascular inflammatory disease, its main pathological feature is lipid deposition in subendothelial space of large- and medium-sized arteries, eventually forming atherosclerotic plaques (Zhu et al., 2018). The formation of atherosclerotic lesions involves the interaction of various cells with cytokines, and the occurrence of inflammatory response exerts a significant effect in promoting the progression of atherosclerosis (Karunakaran et al., 2021). Although statins have obtained considerable improvement in the clinical outcomes of CVD patients, the residual cardiovascular risk is still high (Okuyama et al., 2015). Therefore, a better understanding of the role of key molecules in the pathogenesis of atherosclerosis is important for developing new promising strategies for the prevention and treatment of atherosclerotic CVD.

Tyrosine-protein phosphatase non-receptor type 2(PTPN2), an important member of the protein tyrosine phosphatase (PTPs) family, which was first named T-cell protein-tyrosine phosphatase (TC-PTP) because it was first discovered in T cells (Cool et al., 1989). PTPN2 is an intracellular PTP that consists of a PTP domain and a C-terminus domain (Hongdusit and Fox, 2021). As a dephosphorylation enzyme, PTPN2 can negatively regulate many signaling pathways through dephosphorylation. The biggest manifestation is that PTPN2 can inhibit multiple inflammatory signaling pathways (Meng et al., 2019). In addition, PTPN2 can also regulate many biological processes, including hematopoiesis, cell proliferation, and cell differentiation (Hsieh et al., 2020). Inflammatory response run through all stages of atherosclerosis, such as the formation and rupture of atherosclerotic plaques (Zhu et al., 2018). PTPN2 regulates a variety of pathophysiological processes involving atherosclerosis, including endothelial injury, monocyte proliferation and migration, macrophage polarization, T cell polarization, autophagy, pyroptosis, and insulin resistance (L. Nie et al., 2013; Spalinger et al., 2018; Yang et al., 2022; You et al., 2015). In addition, PTPN2 expression is significantly downregulated in high cholesterol-induced apoE^−/−^ mouse atherosclerotic lesions (Hu et al., 2020).

In this review, we summarized current studies regarding the protective effect and mechanism of PTPN2 in the progression of atherosclerosis-related diseases, which may prove the potential value of PTPN2 in the treatment of atherosclerosis and a basis for further investigation on PTPN2 and atherosclerosis.

## 2 Structure of PTPN2

**FIGURE 1:**
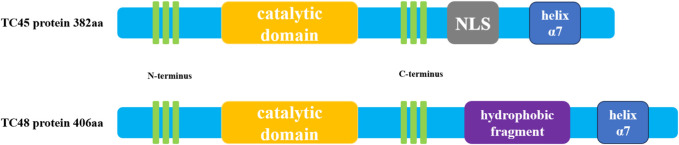
Schematic diagram of human PTPN2 structure. The human PTPN2 gene expresses two protein forms, TC45 and TC48. Both proteins contain a catalytic region, and the C-terminal domain of the protein contains a helix α7 structure. The biggest difference between the two is that the C-terminal domain of TC45 contains an NLS sequence that localizes it in the nucleus, while the C-terminal domain of TC48 contains hydrophobic fragments that localize it in the cytoplasm.

The structure of PTPs contains a conserved catalytic domain and N- or C-terminus non-catalytic fragments (Sharma et al., 2021). PTPs regulate a variety of biological processes such as cell growth, proliferation, migration, differentiation, and apoptosis by catalyzing the dephosphorylation of protein tyrosine phosphorylated residues (Tonks and Neel, 1996). PTPs include two types of members, receptor-like PTP and intracellular PTP (Du and Grandis, 2015). PTPN2 is a typical intracellular PTP, which was first discovered in human T cells and cloned from the T cell cDNA library (Cool et al., 1989). The human PTPN2 gene is mapped on the 18P11.2-P11.3 region (HG19:CHR18:12,785,481-12,884,334), and the mouse PTPN2 gene is also mapped on the homologous region of chromosome 18 (MM9: CHR18: 67825155-67884275) (Sakaguchi et al., 1992). There are four isoforms of PTPN2 (PTP-S1, PTP-S2, PTP-S3 and PTP-S4) in rat cells, of which PTP-S2 (TC45) and PTP-S4 (TC48) are the predominant forms (Chen et al., 2021). In rat DNA, 57 bases (19 amino acids) at the 5′end of exon E were excised using an internal receptor site consistent with the eukaryotic 3′splicer site, resulting in the PTP-S1 and PTP-S3.3′splicer site sequence AG, which is highly conserved in rat DNA and lost in human DNA. Thus, there are only two isoforms in human cells: TC45 (PTP-S2) and TC48 (PTP-S4) (Reddy and Swarup, 1995). Because the mRNA of PTPN2 is selectively spliced, the C-terminal regions of TC45 and TC48 differ in size, hydrophobicity, and function. Three transcripts composed of 10 exons encode a product of 387 amino acids (aa), resulting in a 45.2 kDa protein, which is human cell TC45 (NM_080422.2) (Muppirala, Gupta and Swarup, 2013). A unique exon at the 3′terminal of TC45 encodes 6 hydrophilic amino acids, resulting in TC45 lacking a hydrophobic fragment (residues 382-418). In addition, the C- terminal of PTPN2 (residues 350-358 and 377-381) contains a Nuclear localization sequence (NLS), so TC45 localizes to the nucleus (Tillmann et al., 1994). TC48(NM_002828.3) is a 415aa product encoded by nine exons, producing a 48.5 kDa protein (Muppirala et al., 2013). The alternative splicing event results in the formation of a transcript of TC48 with the penultimate exon and 3′intron extension. Therefore, 6 amino acids at the C- terminal of TC45 were replaced by 34 amino acids in TC48 comprising 19 hydrophobic residues. So TC48 is localized to the endoplasmic reticulum and requires detergent treatment for extraction (Cool et al., 1989; Lorenzen et al., 1995). In mouse cells, TC45(NM_008977.3) consists of 10 exons encoding a 382 AA product to produce a 44.5 kDa protein, and TC48(NM_001127177.1) consists of 9 exons encoding a 406 AA product to produce a 47.4 kDa protein (Muppirala et al., 2013). The residue sequence conservation of mouse and human TC45 is 78.5%, while that of TC48 is 73.6% (Muppirala et al., 2013). The conserved catalytic domain of PTPN2 plays a physiological role in its family, and its C- terminal fragment may interact with other proteins and have targeted functions. The C- terminal domain of PTPN2 is of great importance to its localization and regulatory function because the autoregulatory sites in the non-catalytic fragment of PTPN2 C- terminal have reversible intramolecular interactions with the catalytic domain to regulate the activity of PTPN2(Ylilauri et al., 2013). Some studies have determined the crystal structure of PTPN2 and found that PTPN2 has a helix α7 at its C- terminal. Cutting the helix α7 will reduce the catalytic efficiency of PTPN2, indicating that the structure outside the conservative catalytic domain, such as helix α7, can also regulate the catalytic activity of PTPN2(Singh et al., 2021). Cys216 in PTPN2 corresponds to an active site residue of PTP, and Cys216 has an affinity for the phosphotyrosine residue of the substrate (Nian et al., 2022). Mutation of the Cys residue in this active site renders the PTPase catalytically inactive but still can bind to substrates (Reiterer et al., 2020; Mattei et al., 2021). This indicates that the function of PTPT2 can be regulated not only by changing the conserved catalytic domain of PTPN2 but also by changing the structure outside the conserved catalytic domain. Taken together, understanding the structure features and posttranslational modifications of PTPN2 can help to provide a better understanding of its biological functions in cardiovascular development and disease. The structure diagram of PTPN2 is shown in Figure 1.

## 3 Tissue and intracellular distribution of PTPN2

PTPN2 is more abundant in cells involved in the regulation of inflammation, such as vascular endothelial cells (VECs), monocytes, macrophages, B lymphocytes, T lymphocytes, and other cells (Wiede et al., 2020; Spalinger et al., 2021). In addition, PTPN2 is also highly expressed in the gastrointestinal tract, liver, lung, and brain (Wang et al., 2018). Therefore, deletion of PTPN2 often leads to the development of inflammation-related diseases such as Crohn’s disease, hepatitis, diabetes, and atherosclerosis (Spalinger et al., 2020; Sabev et al., 2021; Zhu et al., 2021). Due to alternative splicing of mRNA, PTPN2 is normally spliced into two isoforms. The C-terminus of TC45 lacks a hydrophobic fragment (residues 382-418), allowing it to localize to the nucleus, while the C-terminus of TC48 has 19 hydrophobic residues, allowing it to localize to the endoplasmic reticulum (Tiganis et al., 1998). In addition, TC45 can also localize to the cytoplasm and cell membrane, and TC48 is also localized to the endoplasmic reticulum-Golgi intermediate compartment (Lorenzen et al., 1995). TC45 can shuttle between the nucleus and cytoplasm and dephosphorylate plasma membrane receptors, thereby regulating various signaling pathways and biological processes. However, it is unknown whether TC48 can also play the same regulatory role.

## 4 PTPN2 and inflammatory signaling

Atherosclerosis has always been considered a chronic inflammatory disease, and its occurrence and development are closely related to an inflammatory response (Mendez-Barbero et al., 2021). Stimulating inflammatory cells and immune cells to produce inflammatory signals promotes the development of atherosclerosis.

Firstly, mitogen-activated protein kinase (MAPK) can activate transcription factors-associated inflammation, such as nuclear factor kappa-B (NF-κB), signal transducer and activator of transcription (STAT)1, and STAT3 by stimulating extracellular regulated protein kinases 1/2 (ERK1/2) and p38MAPK signaling (Bode et al., 2012; Cao et al., 2021). Meanwhile, activation of p38MAPK upregulates the expression of inflammatory cytokines such as tumor necrosis factor -α(TNF-α)and interleukin −1(IL-1) (Nie et al., 2020). The secretion of inflammatory factors by macrophages further promotes endothelial dysfunction and damage, and promote the development of atherosclerosis (Xu et al., 2019). Activation of MAPK in macrophages is significantly increased when humans carry the PTPN2 gene variant: SNP; rs1893217; A>G (Scharl et al., 2012). After the knockdown of PTPN2, the phosphorylation of p38MAPK and ERK1/2 is significantly reduced (Scharl et al., 2011; Moron et al., 2013).

Secondly, after knocking out PTPN2, the expression of inflammatory cytokines such as NF-κB, TNF-α, and IL-6 induced by the MAPK signaling pathway in macrophages is also significantly reduced, and the levels of phosphorylated STAT1 and STAT3 are also significantly reduced (van Vliet et al., 2005; Scharl et al., 2010). In addition, Studies have shown that IFN-γ-induced phosphorylation of STAT1/3 and IFN-γ-induced expression of vascular cell adhesion molecule-1(VCAM-1), monocyte chemoattractant protein-1 (MCP-1), and IL-6 are significantly reduced when PTPN2 expression and activity are increased in macrophages, also inhibiting the activation of p38 MAPK(Scharl et al., 2010; Moron et al., 2013). When the human body carries the PTPN2 gene variant (SNP; rs1893217; A>G), the secretion of IFN-γ also increases (Scharl et al., 2012). A deficiency of PTPN2 leads to aggravation of IFN-γ-induced inflammatory response in macrophages (Elvira et al., 2022).

Thirdly, in macrophages, knockdown of PTPN2 results in a marked increase in the phosphorylation of JAK1, JAK3, and downstream STAT1 and STAT. In PTPN2-deficient mice, the STAT1/STAT3 signaling is activated in CD4^+^ T cells, which promotes the polarization of Type 1/17 helper T (Th1/Th17) cells, resulting in systemic inflammation in mice (Spalinger et al., 2016).

Lastly, Downregulation of PTPN2 expression in macrophages enhances IL-6 secretion (Hamel-Cote et al., 2019). Activation of PTPN2 in macrophages inhibits platelet-activating factor (PAF)-mediated activation of transcription factors such as activator protein-1 (AP-1), CCAAT/enhancer binding protein β(C/EBPß), STAT5, and NF-κB, which in turn inhibits the activation of the IL-6 promoter, results in downregulation of IL-6 expression, ultimately reduces inflammatory response (Hamel-Cote et al., 2019).

PTPN2 has a significant anti-inflammatory effect, which can inhibit or reduce the occurrence of inflammation by regulating related signaling pathways, and then reduce the occurrence and development of atherosclerosis.

## 5 Role of PTPN2 in atherosclerosis

The occurrence and development of atherosclerosis involve multiple etiologies, such as endothelial injury, monocyte proliferation and migration, macrophage differentiation, T-cell polarization, autophagy, pyroptosis, and insulin resistance. Multiple studies have found that PTPN2 may inhibit atherosclerosis through the following mechanisms.

### 5.1 PTPN2 attenuates endothelial injury

After vascular endothelial injury, VECs secretes vascular endothelial growth factor (VEGF), which binds to vascular endothelial growth factor receptor 2(VEGFR2) on VECs to promote receptor dimerization and phosphorylation, and then activates downstream signaling pathways, which can promote the proliferation and migration of VECs, increase microvascular permeability, and promote the formation of new blood vessels *in vivo*, tissue adhesion, then lead to atherosclerosis (Shibuya, 2015; Karaman et al., 2018). Studies have shown that the knockdown of PTPN2 in human umbilical vein endothelial cells (HUVECs) significantly increases the proliferation and migration of VECs induced by VEGF, and undergoes significant morphological changes, VEGF can also induce angiogenesis *in vitro*. In three-dimensional cultures of HUVECs spheroids, activation of PTPN2 by integrinα1(ITGA1) significantly inhibited *in vitro* angiogenesis (Mattila et al., 2008). In addition, PTPN2 phosphatase can bind to VEGFR2 kinase and subsequently promote the dephosphorylation of VEGFR2 and inhibit its signal transduction. In HUVEC, silencing of PTPN2 with siRNA significantly enhances VEGF-induced phosphorylation of p44/42 MAPK and protein kinase B (AKT) and also increases phosphorylation of VEGFR2 to further promote VECs damage and cytokine release (L. Nie et al., 2013). Angiogenin-1 (Ang-1) is the activating ligand of endothelial cell-specific receptor tyrosine kinase 2(Tie-2). In adult blood vessels, Ang-1/Tie-2 signaling reduces endothelial hyperpermeability and inflammation by inhibiting Occludin tyrosine phosphorylation and promoting the interaction of Occludin with ZO-1(Y. Shi et al., 2019). In human brain microvascular endothelial cells (HBMECs), knockdown of PTPN2 significantly elevates the levels of Occludin tyrosine phosphorylation, which in turn leads to a significant reduction in the barrier protection effect of Ang-1 on cells, then increases endothelial cell permeability and inflammation, and ultimately leads to endothelial injury (Siddiqui et al., 2015). PTPN2 reduces VECs injury and inflammatory response by regulating VEGF/VEGFR2 and Ang-1/Tie-2 signaling. Therefore, PTPN2 may act as a regulatory target of atherosclerosis and exert an anti-atherosclerotic effect.

### 5.2 PTPN2 inhibits monocyte proliferation and migration

Vascular endothelial injury and dysfunction can lead to the release of various cytokines and adhesion molecules from VECs, which induces the recruitment of monocytes and other inflammatory cells to the subendothelial layer, which is the initiating link of atherosclerosis (Liang et al., 2020). When PTPN2 is knocked out in monocytes, the expression of VCAM-1, MCP-1, and IL-6 induced by IFN-γ is significantly upregulated, and the overexpression of VCAM-1 and MCP-1 promotes the recruitment of monocytes to the injured vascular endothelium (Scharl et al., 2010; Moron et al., 2013). The EDU assay found that the deficiency of PTPN2 significantly promotes the proliferation of monocytes, and also promotes the proliferation of HUVECs after the subsequent co-incubation of monocytes with HUVECs. In addition, transwell migration and invasion assays showed that the knockdown of PTPN2 in monocytes also enhances cell migration and invasion (Hu et al., 2020). These results suggest that a deficiency of PTPN2 promotes monocyte proliferation, migration, and invasion. Therefore, PTPN2 may function as anti-atherosclerosis.

### 5.3 PTPN2 inhibits macrophage polarization toward M1

Macrophages are abundant in the process of atherosclerotic lesions, and their major types include pro-inflammatory (M1) macrophages and anti-inflammatory (M2) macrophages (Jinnouchi et al., 2020). M1 macrophages can secrete a variety of pro-inflammatory cytokines, such as cytokines TNF-α, IL-1α, IL-1β, IL-6, IL-12, IL-15, IL-23, chemokines CXCL9, CXCL10, and cell mediators ROS and NO(Shapouri-Moghaddam et al., 2018). These may contribute to persistent inflammation around atherosclerotic plaques, recruitment of inflammatory cells, and plaque formation (Farias-Itao et al., 2022). Studies have shown that the expression of PTPN2 in monocytes is significantly downregulated in the apoE^−/−^ inflammatory mouse atherosclerosis model (Yang et al., 2022). Deletion of PTPN2 in monocytes induces their transformation into M1-type macrophages, promotes the secretion of IL-12 and IL-1β, promotes cell proliferation and migration, and increases p65, p38, and STAT3 phosphorylation (Y. Li et al., 2018). Meanwhile, the expression of IL-6, TNF-α, and inducible nitric oxide synthase (iNOS) associated with M1 polarization is significantly upregulated in PTPN2-deficient cells (Hu et al., 2020). When PTPN2 is overexpressed, it can block the inflammatory response of macrophages by mediating the dephosphorylation of p65/p38/STAT3 (Hu et al., 2020). IL-4 induces M2 polarization in macrophages by interacting with its receptor IL-4Rα(L. Shi et al., 2021). IL-6 can promote the expression of IL-4Rα, which in turn promotes IL-4-induced macrophage polarization to M2. Knockdown of PTPN2 in macrophages results in downregulation of IL-6R expression, which in turn inhibited the signaling cascade caused by IL-6 binding to IL-6R, failing M2 macrophage polarization, while the levels of M1 macrophage markers are significantly increased, exacerbating the cellular inflammatory response (Spalinger et al., 2022). These results suggest that PTPN2 inhibits the inflammatory response of atherosclerosis by inhibiting the polarization of macrophages to M2, thus, PTPN2 may play an important role in regulating macrophage polarization and inhibiting atherosclerosis.

### 5.4 PTPN2 inhibits T cell polarization

T cells enter the vessel wall by a mechanism similar to that of monocytes and are subsequently activated upon antigenic stimulation to produce inflammatory cytokines, which further amplify the inflammatory response and aggravate the progression of atherosclerosis (Saigusa et al., 2020). Th1 cells can secrete IFN-γ, TNF-α, IL-4, IL-5, and IL-13, mediate macrophage activation and eosinophils participate in inflammatory response (Ali et al., 2020). Th2 cells may be a pro-atherosclerotic risk factor in the context of hypercholesterolemia (Z. Li et al., 2022; Taleb, 2016). Regulatory T cells (Treg) usually suppress Th1 and Th2 pathological responses and can produce a large amount of transforming growth factor-β (TGF-β) and IL-10, which can inhibit the formation of atherosclerosis (Shapouri-Moghaddam et al., 2018; Y. N; Wang et al., 2021). The new view is that the formation and development of atherosclerotic plaques are caused by the imbalance of pathogenic Th1 cells, Th2 cells, Th17 cells, and Treg (Saigusa et al., 2020). After the isolation of PTPN2-deficient CD4^+^ T cells in the diabetic mouse model, they tend to polarize into Th1 cells, and some of the formation of Th1 cells is due to the enhanced STAT1 signal after PTPN2 knockout (J. Gao et al., 2022; Wiede et al., 2019). The levels of PTPN2 in visceral adipose tissue (VAT) of diabetic apoE−/− mice are significantly downregulated, which leads to pro-inflammatory polarization of T cells in VAT, resulting in the inflammatory response and instability of atherosclerotic plaques (Wiede et al., 2019). The deficiency of PTPN2 in VAT leads to T cell polarization toward Th1 and Th17 cells but not toward Treg (Xue et al., 2022), which results in the imbalance of the Th1, Th17, and Treg ratio (Flosbach et al., 2020). However, PTPN2 overexpression in VAT increases the levels of Treg. Thus, the ratio of Th1/Treg, Th2/Treg, and Th17/Treg is decreased, which alleviates the body’s inflammatory response and ultimately stabilizes the atherosclerotic plaque (Li et al., 2018; Zhang et al., 2018).

In PTPN2-LckCre mice, Treg levels are significantly decreased, while Th1 and Th17 levels are significantly increased, and increased levels of Th1 and Th17-related genes are detected in biopsies from areas of inflammation (Spalinger et al., 2015; Svensson et al., 2019). Moreover, clinical data show that Th17 and IL-17 account for an increased proportion of coronary heart disease and atherosclerosis, and are positively correlated with disease severity (Esmailbeig and Ghaderi, 2017). IL-2 is a necessary cytokine for Treg activation and survival (Abbas et al., 2018). When the PTPN2 gene is deficient, IL-2R signaling in CD4^+^ T cells is inhibited, resulting in a decrease in the sensitivity of CD4^+^ T cells to IL-2, and activation of the Treg is inhibited (Kasahara et al., 2014). Therefore, PTPN2 may serve as an important target for regulating T cell polarization, thereby protecting atherosclerosis.

### 5.5 PTPN2 promotes autophagy

Autophagy has a protective effect on atherosclerosis by inhibiting inflammation and apoptosis, promoting cholesterol efflux, and reducing lipid deposition (Qiao et al., 2021). Autophagy dysfunction promotes the occurrence and development of atherosclerosis (Miao et al., 2020; Robichaud et al., 2021). Atherosclerosis is closely associated with VECs, macrophages, and VSMCs. Impaired autophagy in VECs aggravates atherosclerosis (Wei et al., 2021). Autophagy in macrophages inhibits the formation of foam cells and inhibits the development of atherosclerosis (Y. Cao et al., 2019; Zhang et al., 2021). Promoting autophagy in VSMCs can protect VSMCs under noxious stimuli and play an anti-atherosclerotic role (Grootaert et al., 2018; Shan et al., 2021). The transcriptional enhancer STAT3 of autophagy-related genes in the nucleus performs anti-autophagy by upregulating the expression of negative regulators of autophagy such as B-cell lymphoma-2(BCL2), BCL2L1, myeloid cell leukemia-1 (MCL1), phosphoinositide-3-kinase regulatory subunit 1(PIK3R1)/p55α and PIK3R1/p50α, or by downregulating the expression of essential autophagy genes such as Beclin1 and PIK3C3 (You et al., 2015). STAT3 is generally located in the cytoplasm and can only be transferred to the nucleus when it is dimerized after phosphorylation activation (Liu et al., 2021). PTPN2 is a negative regulator of the JAK/STAT signaling and inhibits its activation and transfer to the nucleus by dephosphorylation of several of its members, such as JAK1/3 and STAT1/3. In macrophages, knockdown of PTPN2 significantly enhances the activation of JAK-STAT, whereas when cells were treated with a JAK inhibitor, the activation of JAK-STAT caused by PTPN2 deletion was significantly inhibited and the phosphorylation levels are significantly reduced (Spalinger et al., 2021). The same conclusion is obtained in PTPN2-LysMCre mice. Deletion of PTPN2 significantly enhanced the activity of the STAT3 transcript, which in turn led to autophagy impairment in macrophages (Zhu et al., 2020). Microtubule-associated protein 1 light chain 3B-Ⅱ(LC3B-II) is a marker of autophagosome formation, and muramyl-dipeptide (MDP) can induce autophagosome formation (Chung et al., 2017). In MDP-treated PTPN2-WT macrophages, the levels of LC3B-II are significantly increased and the levels of p62 are significantly decreased, but in MDP-treated PTPN2-WT macrophages, the levels of LC3B-II are significantly increased, but this effect is blocked in PTPN2 mutant cells (Scharl, et al., 2012). Knockdown of PTPN2 in macrophages also results in downregulated protein expression of autophagy-related molecules, such as Beclin-1, autophagy-related gene (ATG)5/7/12, autophagy-related 16-like 1 (ATG16L1) (Scharl et al., 2012). Deletion of PTPN2 in macrophages leads to increased levels of TNF-α and IFN-γ, these induce the formation of larger LC3B + vacuoles, a marker of dysfunctional autophagosome formation due to defects in the autophagic process (Svensson et al., 2019). Moreover, the deletion of PTPN2 also causes abnormal expression of IRGM, which is also a marker of abnormal autophagy induced by cytokines TNF-α and IFN-γ(Scharl et al., 2012). Therefore, it can be found that PTPN2 inhibits the development of atherosclerosis by regulating the autophagy of macrophages. However, the role of PTPN2 in regulating autophagy in VECs and VSMCs has not been reported and needs further study.

### 5.6 PTPN2 inhibits pyroptosis

Pyroptosis is a process in which the NLRP3 inflammasome is activated and then promotes the release of inflammatory factors such as IL-1β and IL-18 (Gaul et al., 2021). Pyroptosis promotes VECs damage and foam cell formation, and leads to defective reverse cholesterol transport, thereby promoting the development of atherosclerosis (Opoku et al., 2021). It has been reported that PTPN2 regulates pyroptosis from two aspects. On the one hand, PTPN2 negatively regulates the expression of IL-1β and IL-18 by interfering with the transduction of inflammatory signaling pathways (Spalinger et al., 2018; Wang et al., 2023). On the other hand, PTPN2 inhibits the assembly of the NLRP3 inflammasome and prevents the release of IL-1β and IL-18 after its activation (Scharl et al., 2012). The NLRP3 inflammasome is assembled from three parts, NLRP3, ASC, and pro-caspase-1, while the effect of PTPN2 on inflammasome assembly is indirect. First, the loss of PTPN2 leads to increased activity of c-jun-terminal kinase (JNK) and spleen tyrosine kinase (Syk), then JNK and Syk activate the tyrosine phosphatase proline-rich tyrosine kinase (PYK2), and the activated PYK2 induces an increase in apoptosis-associated speck-like protein containing CARD (ASC) tyrosine phosphorylation, the formation of ASC multimers, and the activation of Caspase-1, and finally promotes maturation and release of IL-1β and IL-18 (Spalinger et al., 2018; Spalinger et al., 2020). In macrophages with high PTPN2 expression, the expression of IL-1β and IL-18 is significantly downregulated due to the inhibition of inflammasome assembly (Hu et al., 2020). Therefore, PTPN2 attenuates the cellular inflammatory response and inhibits the further release of inflammatory factors and adhesion molecules from macrophages, thereby reducing the damage of VECs and the formation of foam cells (Moore et al., 2009; Bourdeau et al., 2013). PTPN2 inhibits the development of atherosclerosis by inhibiting pyroptosis and thus may serve as a potential regulatory target of atherosclerosis.

### 5.7 PTPN2 reduces insulin resistance

**FIGURE 2:**
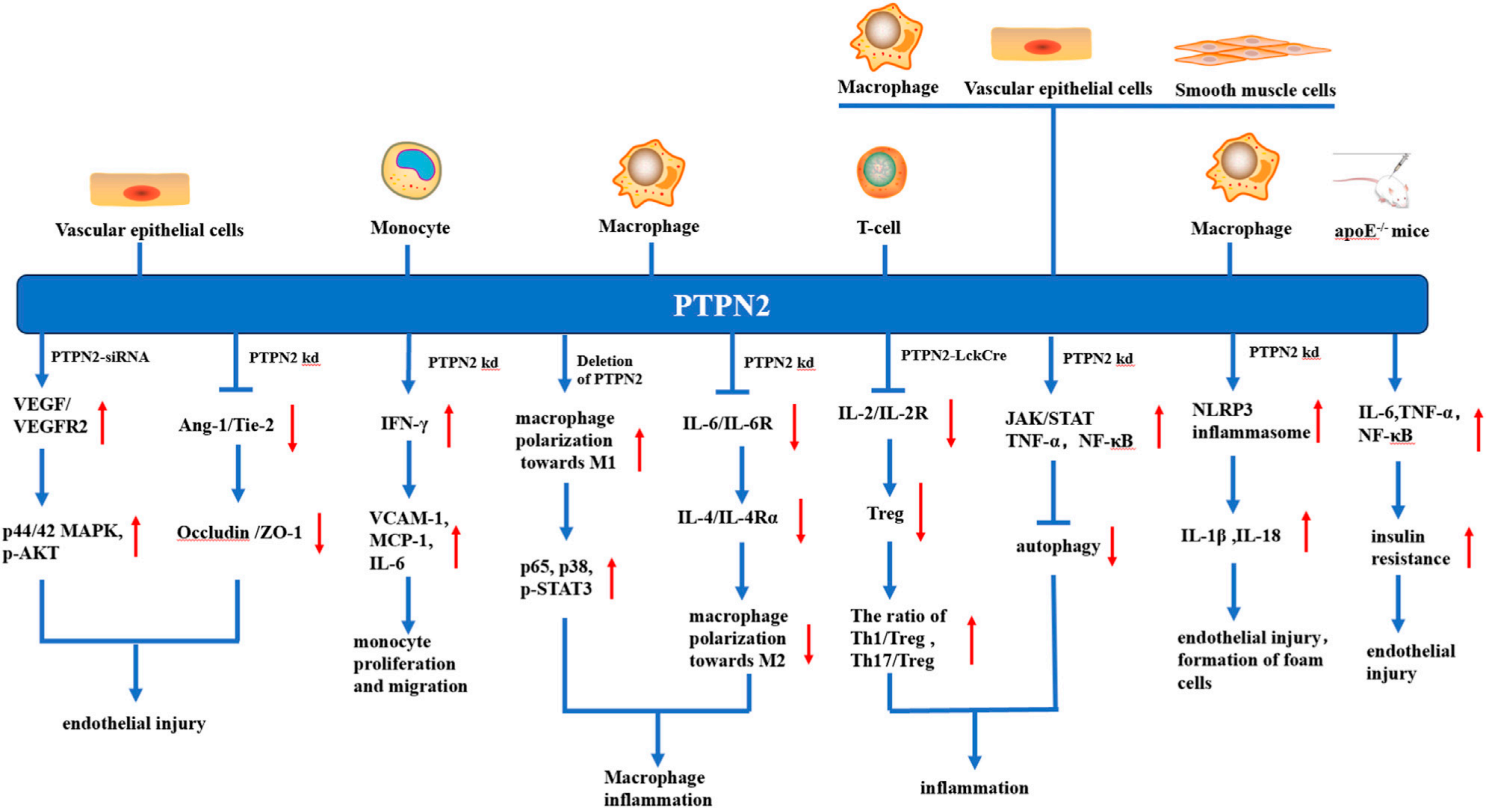
Various anti-atherosclerosis mechanisms of PTPN2 in different kinds of cells. In vascular endothelial cells, knocking down or silting PTPN2 can lead to the binding of VEGF and VEGFR, promote the phosphorylation of p44/42MAPK and AKT, and also weaken the Ang-1/Tie-2 signal, which in turn leads to the reduction of Occludin and ZO-1 junction, both mechanisms ultimately lead to vascular endothelial injury. In monocytes, PTPN2 knockout leads to IFN-γ-induced production of VCAM-1, MCP-1, and IL-6, thereby promoting monocyte proliferation and migration. In macrophages, knockout of PTPN2 causes polarization of macrophages towards M1, which leads to inflammation, and activation of NLRP3 inflammasome, which leads to endothelial damage and foam cell formation. In T-cells, the knockout of PTPN2 will lead to the reduction of Treg, and the imbalance of Th1, Th17, and Treg, thus promoting the occurrence of inflammation. In apoE^−/−^ mice, PTPN2 knockout leads to increased insulin resistance and promotes endothelial injury. In addition, knocking out PTPN2 in macrophages, vascular endothelial cells, and vascular smooth muscle cells also leads to the inhibition of autophagy, which leads to vascular endothelial injury and foam cell formation.

Atherosclerosis is also considered to be a metabolic disease. A large amount of evidence has demonstrated that inflammatory factors released by inflammatory cells lead to aggravation of insulin resistance in the body, and subsequently cause vascular endothelial damage and lipid metabolism disorders, finally accelerating the process of atherosclerosis (Di Pino and DeFronzo, 2019). A recent study showed that apoE^−/−^ mice with combined hyperglycemia and hypercholesterolemia exhibited severe insulin resistance accompanied by a markedly downregulated expression of PTPN2(Y. Li et al., 2019). Overexpression of PTPN2 significantly reduces the levels of NF-κB, TNF-α, IL-6, and the inflammatory response in diabetic mice, and then alleviated insulin resistance. Overexpression of PTPN2 also significantly downregulates the expression of monocytes Cell and T-cell chemokine (MCP-1) and adhesion molecule VCAM-1, thus attenuating VECs damage and monocyte migration (Yoo et al., 2018). The STAT signaling pathway regulates various mediators and participates in the secretion of pro-inflammatory factors, and PTPN2 can dephosphorylate STAT1/3 in diabetic mice and inhibit its inflammatory signaling (Kim et al., 2018). Knockout of PTPN2 in apoE^−/−^ mice leads to severe insulin resistance, and then leads to disordered lipid metabolism and atherosclerosis (Gurzov et al., 2015). PTPN2 treatment significantly reduces serum triglyceride, total cholesterol, and low-density lipoprotein-cholesterol levels, and also reduces metabolic disturbances and hyperglycemia in mice (Y. Li et al., 2019; Wiede et al., 2019). PTPN2 reduces insulin resistance by inhibiting the inflammatory response of cells and the body and then plays a role in regulating the lipid metabolism disorder of the body, so it can be used as a therapeutic target for reducing atherosclerosis. The possible targets of PTPN2 are summarized in Table 1, and the related signaling pathways of PTPN2 are shown in Figure 2.

**TABLE 1:** The target genes of PTPN2 and its roles in atherosclerosis.

Targets	Effects	Function	As	Reference
MAPK	Inactivation	Reduce insulin resistance	↓	Moron et al. (2013)
		Reduce macrophages inflammation		
JAK1	Inactivation	Reduce insulin resistance	↓	Zhang et al. (2018)
JAK3	Inactivation	Reduce insulin resistance	↓	Li et al. (2019)
STAT1	Inactivation	Suppresses Th1 cell formation	↓	Spalinger et al. (2016)
STAT3	Inactivation	Promote autophagy	↓	Spalinger et al. (2021)
TNF-α	Inactivation	Reduce insulin resistance	↓	Meng et al. (2019)
		Promote autophagy		
		Monocyte migration		
IFN-γ	Inactivation	Monocyte migration	↓	Moron et al. (2013)
		Promote autophagy		
IL-4	Inactivation	Suppresses VECs injury	↓	Kim et al. (2019)
	Activation	Promote the polarization of macrophages to M2		
IL-6	Inactivation	Reduce macrophages inflammation	↓	Hamel-Cote et al. (2019)
VEGF	Inactivation	Suppresses VECs injury	↓	Mattila et al. (2008)
VEGFR2	Inactivation	Suppresses VECs injury	↓	Nie et al. (2013)
Occludin	Inactivation	Suppresses VECs injury	↓	Shi et al. (2019)
IL-6R	Activation	Promote the polarization of macrophages to M2	↓	Spalinger et al. (2022)
IL-2R	Activation	Promote Treg formation	↓	Kasahara et al. (2014)
MDP	Activation	Promote autophagy	↓	Scharl et al. (2012)
Beclin −1	Activation	Promote autophagy	↓	Scharl et al. (2012)
ATG5	Activation	Promote autophagy	↓	Scharl et al. (2012)
ASC	Inactivation	Suppresses NLRP3 inflammasome formation	↓	Spalinger et al. (2018)
IL-1β	Inactivation	Suppresses NLRP3 inflammasome formation	↓	Spalinger et al. (2018)
IL-18	Inactivation	Suppresses NLRP3 inflammasome formation	↓	Spalinger et al. (2018)

↓, inhibitory effect; AS, atherosclerosis.

## 6 Therapeutic strategies to promote PTPN2 expression

PTPN2 plays a regulatory role in many signaling pathways and biological processes, and activation of PTPN2 plays an important role in diseases such as atherosclerosis, tumors, inflammatory bowel diseases, autoimmune diseases, and diabetes. Therefore, PTPN2 is an important target of drug development. Studies have found that there are some small molecule drugs and proteins that can activate PTPN2. The N-terminus of TC45 protein inhibits its activation by binding to the C-terminus, while the α1-Cyt in the ITGA1 structure interacts with the N-terminus of TC45 and competes with the C-terminus of TC45 to bind to the N-terminus, thereby relieving autoinhibition and activating TC45 (Singh et al., 2022). Activation of TC45 by α1-Cyt inhibits EGF-induced EGFR phosphorylation and reduces cell proliferation and malignant cell growth (Mattila et al., 2005). Spermidine and mitoxantrone can compete with α1-Cyt for binding to the N-terminus site of TC45, indicating that spermidine and mitoxantrone also activate TC45 in the same way as ITGA1 (Ylilauri et al., 2013; Niechcial et al., 2023). Spermidine can inhibit cell proliferation and attenuate RTK signaling in a TC45-dependent manner, and spermidine also can inhibit the activation of IFN-γ-induced downstream signaling by activating PTPN2(Niechcial et al., 2020; Shaw et al., 2021). In adult blood vessels, activation of PTPN2 by Ang-1/Tie-2 signaling promotes the dephosphorylation of Occludin, which in turn promotes the binding of Occludin to ZO-1, thereby regulating endothelial cell permeability and inflammatory responses (Siddiqui et al., 2015). VSL#3 probiotics can increase the enzyme activity and expression of PTPN2, inhibiting its downstream signaling pathway (Krishnan, Penrose, Shah, Marchelletta and McCole, 2016). Activation of PPARδ in the nucleus can form a stable complex with TC45, which can prolong the time that TC45 stays in the nucleus, thereby reducing IL-6-induced insulin resistance, promoting the inactivation of STAT3-suppressor Of cytokine signaling 3(SOCS3) signaling, and alleviating inflammatory response (Mattila et al., 2010). Ruthenium red, MDL-26,630-trihydrochloride, N21 (C15H13N5), and F12 (C30H38N4O2) are also found to be agonists of PTPN2 by high-throughput screening and activate PTPN2 in a concentration-dependent manner (Mattila et al., 2010). In addition, synthetic activators or agonists for specific PTPN2 have not been identified in animal models of atherosclerosis, but further research may be required in the future, and this area of research will certainly facilitate the development of PTPN2 centered therapies that ultimately reduce the risk of atherosclerotic cardiovascular disease.

## 7 Conclusion and future directions

PTPN2 is an important member of the protein tyrosine phosphatase family, which has attracted more and more attention in recent years. PTPN2 mainly inhibits the occurrence and development of atherosclerosis by negatively regulating the expression of downstream target genes and their signaling pathways. These target genes are involved in a series of inflammatory responses, which in turn affect the function of VECs, monocyte proliferation and migration, macrophage polarization, T cell polarization, autophagy, pyroptosis, and insulin resistance, and play an important role in the disease progression of atherosclerosis. In addition, the development of atherosclerosis is closely related to lipid metabolism disorders, cholesterol reverse transport plays a good protective role in atherosclerosis. ABCA1 is a key protein in the reverse cholesterol transport process, which can promote macrophages’ excretion of lipids, thereby inhibiting the development of atherosclerosis. These current studies demonstrate that PTPN2 in macrophages inhibits the development of atherosclerosis by regulating IFN-γ, JAK/STAT1, IL-4/6, and NF-κB-induced inflammation. Our group’s study showed that in macrophages, IFN-γ downregulates the expression of ABCA1 by activating the JAK/STAT1 signaling pathway, thereby promoting the development of atherosclerosis (Hao et al., 2009). Moreover, IL-4/6 and NF-κB can also downregulate the expression of ABCA1 in macrophages and promote the development of atherosclerosis (Ren et al., 2018; Zhao et al., 2021). Therefore, I speculate that PTPN2 may inhibit the progression of atherosclerosis by regulating lipid metabolism in macrophages, but this requires further investigation in the future.

Although abundant data is demonstrating that PTPN2 alleviates insulin resistance in diabetic mice, some studies have shown a conflicting result that activation of PTPN2 inhibits insulin signaling, thereby exacerbating the development of diabetes (Tiganis, 2013; Wang et al., 2021). Numerous studies have shown that the deletion of PTPN2 upregulates the expression of inflammatory factors such as TNF-α, NF-κB, IFN-γ, and IL-6 in THP-1 macrophages and promotes the activation of MAPK. However, in RAW264.7 macrophages, the deletion of PTPN2 downregulates the expression of inflammatory factors and MAPK phosphorylation (Ha Thi et al., 2016). Therefore, further studies of PTPN2 are required to clarify its exact role in atherosclerosis in the future. Although numerous studies have demonstrated the beneficial effects of high PTPN2 expression, effective strategies to promote PTPN2 expression require further exploration. In addition, more work is needed to elucidate how PTPN2 can be most efficiently targeted through transcriptional/post-transcriptional regulation or post-translational modification. In addition, several key questions remain to be answered in future studies: 1) Are there other target genes of PTPN2 that can influence the progression of atherosclerosis? 2) Which genes can regulate the expression of PTPN2 and its downstream signaling pathways? 3) Do the two isoforms of PTPN2, TC45 and TC48, respectively regulate different signaling pathways due to their different intracellular localization? Are the two isoforms consistently expressed in various diseases? 4) What is the specific mechanism by which PTPN2 reduces serum triglyceride, total cholesterol, and LDL-cholesterol levels in diabetic mice? 5) In addition to affecting the polarization of macrophages by affecting the occurrence of inflammatory responses, does PTPN2 also affect the uptake and excretion of lipids by macrophages to inhibit the development of atherosclerosis? 6) PTPN2 can promote the autophagy of macrophages, but does it promote the autophagy of VSMCs and VECs? Answers to such questions will undoubtedly provide unique insights into the role of PTPN2 in atherosclerosis and make PTPN2 an attractive therapeutic target aiming at reducing atherosclerosis.

## Figures and Tables

**Table:** 

**PTPN2**	Tyrosine-protein phosphatase non-receptor type 2
**CVD**	Cardiovascular disease; As, Atherosclerosis
**VECs**	vascular endothelial cells
**TCPTP**	T-cell protein-tyrosine phosphatase
**MAPK**	Mitogen-Activated Protein Kinase
**JAK/STAT**	Janus Kinase/signal transducer and activator of transcription
**IFN-γ**	γ Interferon
**TNF-α/NF-κB**	Tumor Necrosis Factor -α/nuclear factor kappa-B
**IL-4**	interleukin-4
**ERK1/2**	extracellular regulated protein kinases1/2
**VSMC**	vascular smooth muscle cells
**Th1/Th17**	Type 1/17 helper T cells
**NO**	Nitric oxide
**ROS**	reactive oxygen species
**LDL**	Low-density lipoprotein
**VCAM-1**	vascular cell adhesion molecule-1
**MCP-1**	monocyte chemoattractant protein-1
**PAF**	platelet activating factor
**AP-1**	activator protein-1
**C/EBPß**	CCAAT/enhancer binding protein β
**VEGF**	vascular endothelial growth factor
**VEGFR2**	Vascular Endothelial Growth Factor Receptor 2
**HUVECs**	Human umbilical vein endothelial cells
**ITGA1**	integrinα1
**AKT**	protein kinase B
**Ang-1**	Angiogenin-1
**Tie-2**	endothelial cell-specific receptor tyrosine kinase 2
**ZO-1**	zonule occluden 1
**HBMECs**	Human brain micro-vascular endothelial cells
**CXCL9**	chemokines Chemokine (C-X-C motif) ligand 9
**iNOS**	Inducible Nitric-Oxide Synthase
**Treg**	regulatory T cells
**TGF-β**	transforming growth factor-β
**VAT**	visceral adipose tissue
**BCL2**	B-cell lymphoma-2
**MCL1**	myeloid cell leukemia-1
**PIK3R1**	Phosphoinositide-3-Kinase Regulatory Subunit 1
**LC3B-II**	microtubule-associated protein 1 light chain 3B-Ⅱ
**MDP**	muramyl-dipeptide
**ATG**	autophagy-related gene
**ATG16L1**	autophagy-related 16-like 1
**LRP3**	nod-like receptor protein 3
**JNK**	c-JunN-terminal kinase
**Syk**	spleen tyrosine kinase
**PYK2**	Proline-rich tyrsosine kinase 2
**ASC**	apoptosis-associated speck-like protein containing CARD
**RTK**	receptor tyrosine kinase
**PPARδ**	peroxisome proliferator-activated receptor δ
**SOCS3**	suppressor Of cytokine signaling 3
